# Progression of Untreated Mild Aortic Valve Disease in Patients Undergoing Rheumatic Mitral Valve Surgery: A Meta-Analysis of Reconstructed Time-to-Event Data

**DOI:** 10.3390/jcdd12110426

**Published:** 2025-10-28

**Authors:** Chong Luo, Xiaoli Qin, Honghua Yue, Weitao Liang, Zhong Wu

**Affiliations:** Department of Cardiovascular Surgery, West China Hospital, Sichuan University, Chengdu 610041, China; a18583906790@163.com (C.L.); qinxiaoli1993@hotmail.com (X.Q.); yuecqmu@163.com (H.Y.); liang-weitao@hotmail.com (W.L.)

**Keywords:** aortic stenosis, mitral valve surgery, rheumatic heart disease, long-term outcome, meta and landmark analysis

## Abstract

(1) Background: Concomitant mild aortic valve disease is frequently found in patients undergoing rheumatic mitral valve surgery. To date, only a limited number of single-center studies have specifically addressed the untreated baseline aortic valve disease long-term progression and reoperation rate. Thus, we conducted a meta and landmark analysis to systematically review the issue. (2) Methods: This study investigated the long-term prognostic of baseline mild aortic valve disease in patients undergoing rheumatic mitral valve surgery, based on evidence from PubMed, Embase, Cochrane Library, and Web of Science databases. (3) Results: Meta analysis revealed that patients with mild aortic valve disease had a higher risk of disease progression, with a 3.3-fold risk in the 0–5-year follow-up, which jumped to a hazard ratio of 6.42 in longer-term follow-up (5–25 years). Patients with aortic stenosis had an 8.37-fold risk of progression compared with aortic regurgitation and appeared to be poorly related to the time cut-off. Similarly, higher reoperation rates at long-term follow-up were seen in aortic stenosis patients. (4) Conclusions: This study suggests that patients with mild aortic valve disease at baseline have poorer long-term aortic valve-related progression and reoperation rates, especially aortic stenosis. For those with concomitant aortic stenosis, further investigation of the impact of lesion progression is warranted.

## 1. Introduction

Rheumatic heart disease (RHD) is characterized by valvular damage resulting from inflammatory cardiac lesions caused by rheumatic fever. According to a 2019 report, the global prevalence of RHD was 513.68 cases per 100,000 people, with a mortality rate of 3.85 deaths per 100,000 [1]. Among RHD cases, the mitral and aortic valves are the most commonly affected, with the majority of patients (85%) presenting moderate-to-severe mitral valve lesions and 25.9% exhibiting multivalvular involvement [2]. For patients with rheumatic mitral valve disease requiring surgical intervention and coexisting aortic valve lesion, current guidelines recommend aortic valve replacement as a class IIa indication during mitral valve surgery in cases of moderate aortic stenosis, though this recommendation lacks robust clinical evidence (level of evidence C) [3]. To date, limited information is available from only a few single-center studies regarding the progression of aortic valve disease in patients undergoing rheumatic mitral valve surgery who have concomitant mild, unaddressed aortic valve pathology [4,5,6,7,8,9,10,11]. In light of these gaps, this study focuses on the rheumatic mitral surgery population, employing a meta-analysis of reconstructed time-to-event data to evaluate the long-term outcomes of aortic valve disease in such patients, aiming to provide evidence-based insights for optimizing operative management strategies and determining the optimal timing of interventions.

## 2. Materials and Methods

**Figure 1:**
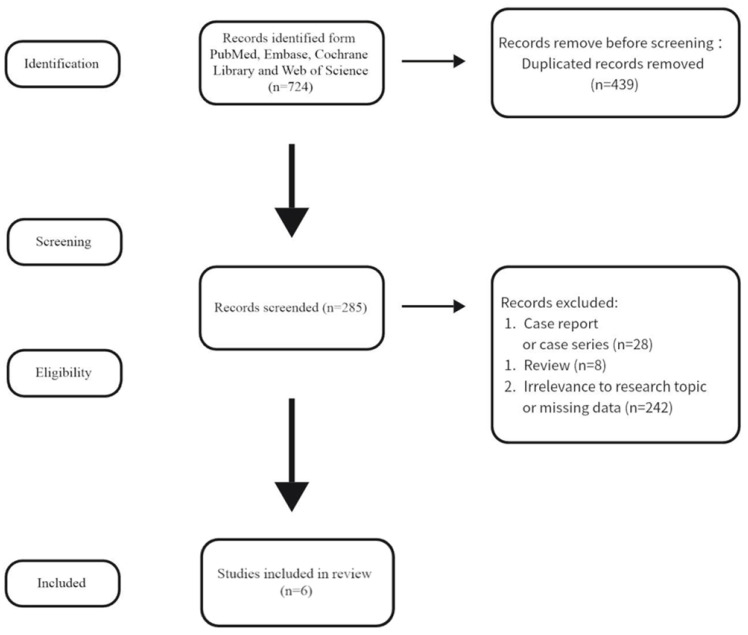
PRISMA flowchart of literature search.

The meta-analysis was performed following the Preferred Reporting Items for Systematic Reviews and Meta-analyses (PRISMA) guidelines [12] (PRISMA Checklist is shown in the Supplementary Materials) and was registered in the International Prospective Register of Systematic Reviews (PROSPERO) database (No. CRD42025641537) (Figure 1).

### 2.1. Search Strategy

A systematic literature review (selecting studies published from 1995 to 2024) was conducted in major databases, including PubMed, Embase, Cochrane Library, and Web of Science. To identify all relevant studies in these databases, the following keywords and medical subject heading terms were used: rheumatic mitral valve disease, mitral valve/surgery, mitral valve repair, mitral valve replacement, aortic valve stenosis, aortic valve insufficiency, aortic valve regurgitation, and aortic valve disease progression.

### 2.2. Selection Criteria

The literature selection criteria for this study were as follows: Observational studies or subgroup analyses of randomized controlled trials (RCTs) investigating the long-term outcomes of mild aortic valve disease (AVD) left untreated during rheumatic mitral valve surgery were included. Eligible participants were adult patients (≥18 years) undergoing rheumatic mitral valve repair or replacement, with baseline AVD meeting guideline-defined mild criteria [13,14]. Although data collection for some included studies spanned an early period with inherent variations in diagnostic precision, this research primarily focuses on the long-term pathological progression of aortic valve disease. By applying consistent diagnostic criteria to evaluate longitudinal pathological changes, we maintain that the findings retain substantial credibility and clinical relevance, while acknowledging the risk of bias [15]. Studies were required to report outcomes such as AVD progression, reoperation rates, or mortality over a postoperative follow-up period of ≥5 years. Exclusions comprised studies involving non-rheumatic mitral valve pathologies, baseline moderate-to-severe AVD, case reports, conference abstracts, or duplicated data. Two investigators independently performed the literature screening, with discrepancies resolved through discussion or arbitration by a third author. The Newcastle–Ottawa Scale was utilized to assess the risk of bias in retrospective cohort studies.

### 2.3. Statistical Analyses

All statistical analyses were conducted using RevMan (version 5.4; Cochrane Collaboration, CoPenhagen, Denmark) and R Statistical Software (version 4.4.1, Foundation for Statistical Computing, Vienna, Austria). For the meta-analysis of AVD progression, hazard ratios (HRs) and 95% confidence intervals (CIs) were derived from published Kaplan–Meier survival curves using the following steps. (1) Data extraction: to reconstruct individual time-to-event data, survival curve coordinates were derived from the web software development by Liu et al. [16]. (2) HR calculation: using a validated Excel spreadsheet, the HR and 95% CI were calculated from the extracted survival probabilities and number of patients at risk using the method of Tierney et al. [17]

Landmark analysis of AVD progression: The pooled HR with 95% CIs and a Kaplan–Meier plot was calculated by R packages survival (version 3.8-3, Terry Therneau, Mayo Clinic, Rochester, MN, USA), survminer (version 0.5.0, Alboukadel Kassambara, Montpellier, France) and dplyr (version 1.1.4, Hadley Wickham et al., Posit PBC, Boston, MA, USA) using previously acquired individual time-to-event data. The landmark plot was completed using the R packages survival (version 3.8-3, Terry Therneau, Mayo Clinic, Rochester, MN, USA) and jskm (version 0.5.11, Jinseob Kim, Zarathu Co., Ltd., Seoul, Korea) as instructed by Caldonazo and Morgan et al. [18,19].

Heterogeneity was assessed using the *χ*^2^ test and quantified by the *I*^2^ statistics, with *I*^2^ > 50% or *p* < 0.05 indicating substantial heterogeneity. Given the broad time span of the literature included in this study and the potential for heterogeneity, the random-effects model was employed for the meta-analysis. As the original articles indeed lacked data such as original IPD, baseline gradients, LV size, age, RHD activity and socioeconomic context, we were unable to conduct targeted adjustments for any specific variables. Therefore, we employed the E Value method to convert confounding bias into an objective, verifiable threshold.

Categorical data are presented as counts and percentages, while continuous data are presented as means ± standard deviations for normally distributed data. Publication bias was assessed using a funnel plot constructed using Egger’s and Begg’s test.

## 3. Results

### 3.1. Study Characteristics

The search strategy retrieved a total of 724 relevant publications, of which 6 studies met the inclusion criteria; no relevant randomized controlled trials were identified after reviewing the literature databases. Figure 1 shows the screening process according to the PRISMA guidelines. The sample size of individual studies ranged from 111 to 1228, and these studies were published between 1999 and 2023. All studies enrolled patients with predominantly rheumatic mitral valve pathology, of whom 1178/3093 (38.1%) had concomitant mild-to-moderate AVD at baseline, including aortic stenosis (AS) and aortic regurgitation (AR). Outcomes focused on longitudinal aortic valve function, including progression to more than moderate AVD and reoperation rates.

### 3.2. Clinical Characteristics

Among the six included studies, four were conducted in upper-middle-income countries with sample sizes ranging from 111 to 1228 patients. The median follow-up duration across studies was 11.54 years (inter-study range: 10.8–13.0 years). Preoperative aortic valve hemodynamic status was systematically documented in all investigations. 4 studies (*n* = 2698 stratified patients by aortic valve pathology, comparing those with mild aortic valve disease (AVD, *n* = 783) against non-AVD (NAVD, *n* = 1915). The remaining two studies focused on functional distinctions between mild AS and AR. Surgical interventions were predominantly mitral valve replacements, complemented by valve repair procedures. Detailed characteristics of the included cohorts are presented in Table 1. The clinical characteristics of the participants could be seen in Table 2.

### 3.3. Worsening of Baseline Aortic Valve Dysfunction Above Moderate Levels

**Figure 2:**
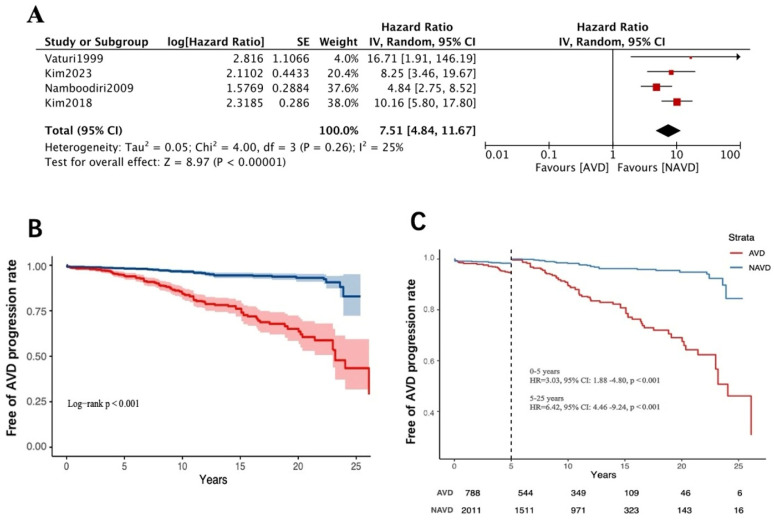
(**A**) Forest plots of the meta-analysis depicting long-term AVD progression rate between AVD and NAVD group [4,5,8,9]. (**B**) Free-of-AVD-progression rate in patients between AVD and NAVD group. Pooled survival curve data were derived from reconstructed event individual time-to-event data from the included literature. (**C**) Landmark analysis of free of AVD progression rate in patients between AVD and NAVD group. AVD group: patients undergoing rheumatic mitral valve surgery, with baseline mild aortic valve disease. NAVD (non-AVD) group: patients undergoing rheumatic mitral surgery, without baseline mild aortic valve disease.

Based on pooled patient data from four studies (*n* = 2799), among the AVD group patients, 20.56% (*n* = 788) experienced aortic valve disease progression over a mean follow-up of 10.98 years, whereas only 2.98% (*n* = 2011) of NAVD group patients (mean follow-up of 11.57 years) showed progression. Our meta-analysis revealed that the AVD group had a significantly higher risk of aortic valve disease progression compared to the NAVD group (HR = 7.51, 95% CI: 4.84–11.67; *p* < 0.001; *I*^2^ = 25%, random-effects model) (Figure 2A). The E-value for the confidence interval lower bound was 9.15 (HR = 7.51, 95% CI: 4.84–11.67).

This indicates that unmeasured confounders would need to increase both exposure likelihood and outcome risk by > 9.15-fold to fully explain the observed association. This exceeds the maximum confounding impact strength of known strong confounders in valvular heart disease, supporting the robustness of the conclusions against potential confounding. Landmark analysis suggested that the rate of lesion progression, despite already showing a substantial elevated risk in the 0–5-year follow-up window (HR = 3.03, 95% CI:1.88–4.80), jumped to a hazard ratio of 6.42 (95% CI:4.46–9.24) in the longer-term follow-up (5–25 years) (Figure 2B,C).

**Figure 3:**
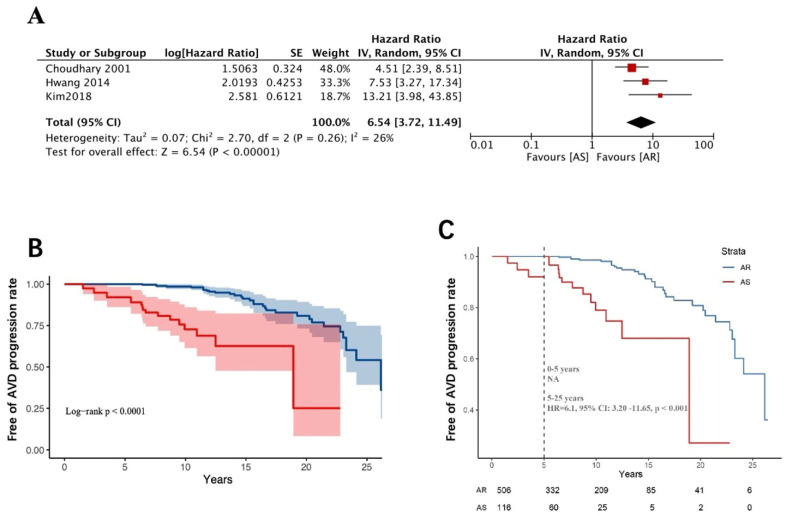
(**A**) Forest plots of the meta-analysis depicting long-term AVD progression rate between AS and AR group [6,8,11]. **(B**) Free-of-AVD-progression rate in patients AS and AR group. Pooled survival curve data were derived from reconstructed event individual time-to-event data from the included literature. (**C**) Landmark analysis of free-of-AVD-progression rate in patients between AS and AR group. AS group: patients undergoing rheumatic mitral valve surgery, with baseline mild aortic stenosis. AR group: patients undergoing rheumatic mitral valve surgery, with baseline aortic regurgitation.

Further subgroup analysis stratified the AVD group into AS and AR. Pooled meta-analysis of survival data from three studies (*n* = 733) demonstrated that patients with AS had a markedly higher risk of aortic valve disease progression than those with AR (HR = 6.54, 95% CI: 3.72–11.49; *p* < 0.001; *I*^2^ = 26%, random-effects model) (Figure 3A). The E-value for the confidence interval lower bound was 6.90 (HR = 6.54, 95% CI: 3.72–11.49).

Begg’s test and Egger’s test indicated no statistically significant publication bias. The landmark analysis suggests the presence of aortic stenosis at baseline, with a faster rate of lesion progression early in the postoperative period, and appears to be poorly related to the time cut-off point. (Figure 3B,C).

### 3.4. Long-Term Follow-Up of Aortic Valve-Related Reoperation Rates

**Figure 4:**
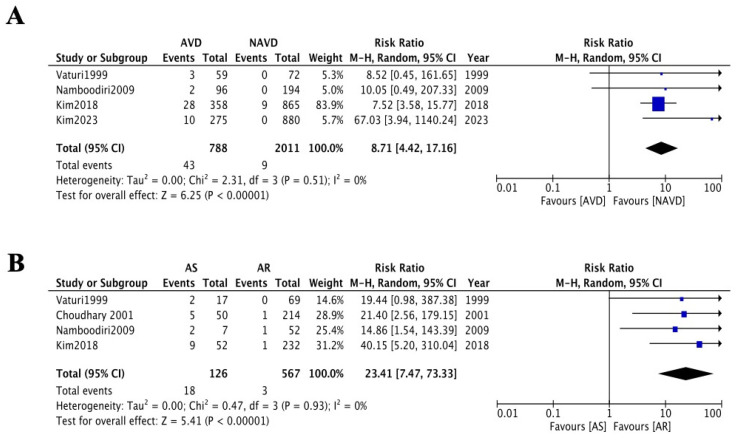
(**A**) Forest plots of the meta-analysis depicting reoperation rate between AVD and NAVD group [4,5,8,9]. (**B**) Forest plots of the meta-analysis depicting reoperation rate between AS and AR group [4,5,8,11].

We conducted a pooled data analysis and meta-analysis of four studies (*n* = 2799) to evaluate the rate of aortic valve-related reoperation after rheumatic mitral valve surgery. In the AVD group (788), 43 patients (5.31%) required reoperation during a mean follow-up of 11.57 years, whereas only 9 patients (0.51%) in the NAVD group (*n* = 2011) underwent reoperation over a mean follow-up of 11.12 years. Meta-analysis demonstrated a significantly higher risk of aortic valve-related reoperation in the AVD group compared to the NAVD group (RR = 8.71, 95% CI: 4.42–17.16; *p* < 0.001; *I*^2^ = 0%, random-effects model, Figure 4A). The E-value for the confidence interval lower bound was 8.3 (RR = 8.71, 95% CI: 4.42–17.16).

Subgroup analysis further stratified the AVD group into AS and AR. A meta-analysis of patient data from four studies (*n* = 787) revealed that the AS subgroup had a significantly higher risk of reoperation compared to the AR subgroup (RR = 23.41, 95% CI:7.47–73.33; *p* = 0.02; *I*^2^ = 77%, random-effects model, Figure 4B). However, this result was accompanied by wide CI (1.34–67.12) and substantial heterogeneity (*I*^2^ = 77%). Sensitivity analysis excluding one outlier study eliminated heterogeneity (*I*^2^ = 0%) but retained a broad confidence interval (RR = 24.28, 95% CI: 6.27–94.02; *p* < 0.001), indicating persistent uncertainty potentially attributable to low reoperation rates and limited subgroup sample sizes. Begg’s test and Egger’s test indicated no statistically significant publication bias.

## 4. Discussion

Rheumatic heart disease remains the predominant acquired valvular cardiovascular disorder in low–middle-income countries and high-risk populations in high-income nations. Long-term follow-up studies over 20 years have reported that rheumatic inflammation can affect all valves [20,21]. However, the mitral valve is most commonly involved, with 25.9% of patients presenting with multivalvular disease [2]. Controversy persists regarding whether concurrent aortic valve replacement should be performed in mild AVD lesion with severe mitral disease. The central issue lies in evaluating the balance between the perioperative risks of concurrent aortic valve replacement (AVR)—including mortality and complication rates—and the long-term complications associated with prosthetic valves (e.g., thrombosis, structural deterioration), against the potential risk of accelerated progression of native aortic valve disease if preserved, which may necessitate reoperation via repeat sternotomy and could delay optimal intervention timing. The critical concern is determining when the cumulative risks of immediate surgical intervention and lifelong prosthetic valve complications outweigh the risks of disease progression in the retained natural valve.

Although current guidelines recommend AVR for patients with moderate aortic stenosis (Class IIa recommendation), no definitive strategies exist for managing mild AVD [3]. Bernal et al. reported outcomes for patients with severe rheumatic mitral valve disease and mild AVD who underwent aortic valve repair: 74.7% required reoperation for aortic valve progression over 22 years [22]. In contrast, long-term follow-up studies of strategies leaving mild AVD untreated reported aortic valve reoperation rates of up to 7.8% [8]. However, these findings derive from single-center retrospective cohorts, limiting their utility for clinical decision-making. This systematic review and meta-analysis aim to evaluate the long-term functional outcomes of the aortic valve in such patients, providing evidence-based insights to guide postoperative management and optimal intervention timing.

From a pathological perspective, persistent rheumatic inflammation leads to valvular surface damage, followed by gradual calcification and leaflet fusion, ultimately resulting in valvular stenosis and regurgitation [23,24]. Nearly one-fourth of patients with rheumatic mitral valve disease also develop involvement of the aortic valve [2]. Vaturi et al. hypothesized that in patients undergoing mitral valve repair or replacement for rheumatic disease, the progression of aortic valve pathology might differ from natural degenerative processes due to chronic hemodynamic and morphological changes following surgery. However, large-scale data supporting these differences remain lacking. Their study specifically noted that patients in the NAVD group demonstrated no significant progression of aortic valve pathology during long-term follow-up [4]. In a 2018 retrospective study by Kim et al. involving a larger cohort, the 20-year freedom from severe AVD progression was 96.5 ± 1.1% in the NAVD group, whereas this rate plummeted to 73.7 ± 4.4% in the AVD group [8]. Our meta-analysis of four studies (*n* = 2799) on the long-term outcomes of rheumatic mitral valve surgery with baseline AVD revealed that NAVD patients exhibited a markedly low risk of AVD progression, aligning with the natural course of degenerative aortic valve pathology [25,26,27]. The AVD group showed a 7.40-fold higher risk of aortic valve disease progression compared to the NAVD group (HR = 7.51, 95% CI 4.84–11.67, Figure 2A). Further analysis of three studies (*n* = 733) demonstrated that patients with AS had a significantly higher risk of AVD progression than those with AR (HR = 6.54, 95% CI: 3.72–11.49, Figure 3A). Through the first attempted analysis of Kaplan–Meier survival curves from published studies, landmark analysis revealed that patients with baseline AVD demonstrated lower progression rates during the early postoperative period (0–5 years) compared to the extended follow-up phase (5–25 years) (Figure 2B,C), whereas those with baseline AS exhibited no temporal threshold for stratifying disease progression (Figure 3B,C). This disparity likely stems from distinct pathophysiological mechanisms: AS is characterized by commissural endothelial injury, fibrin deposition, and fibrocalcific fusion, resulting in a reduced valve orifice area and elevated transvalvular pressure gradients [28,29,30]. AR predominantly involves the free edges of the leaflets, characterized by leaflet contracture [31,32]. The two types of AVD have significant differences in their effects on valvular hemodynamic changes and, in addition, the progression of AR is mostly associated with left ventricular diameter or annular dilatation [33]. Mitral valve surgery may mitigate AR progression by improving left ventricular remodeling, whereas the mechanical stress inherent to AS—driven by fixed obstruction—remains unaltered by such interventions. This may underscore distinct pathogenic trajectories in AS, highlighting the need for future investigations to prioritize mechanistic exploration of hemodynamic perturbations and progressive valvular remodeling patterns specific to AS. Kim et al. further identified baseline aortic valve peak pressure gradient as a critical predictor of AVD progression: patients with a preoperative gradient >11 mmHg faced a significantly elevated risk of disease advancement during long-term follow-up [9].

As well as the progression rate of AVD during long-term follow-up mentioned above, a second critical concern for these patients is the reoperation rate due to AVD progression. Our meta-analysis of four studies (*n* = 2799) revealed that during long-term follow-up (>10 years), AVD group patients had a significantly elevated risk of requiring reoperation for aortic valve replacement (RR =8.71, 95% CI: 4.42–17.16, Figure 4A). Similarly, in a meta-analysis of four studies (*n* = 787) comparing patients with AS and AR, those with baseline mild AS had a higher likelihood of reoperation due to disease progression compared to those with mild AR (RR = 23.41 95% CI: 7.47–73.33, Figure 4B). Although current evidence in Figure 3 suggests faster progression of AS versus AR, the high heterogeneity and wide confidence intervals in this analysis preclude definitive conclusions regarding long-term reoperation risk in mild AS patients, necessitating future large-scale validation to establish reliable evidence. Nevertheless, it is noteworthy that the absolute reoperation rate for baseline AS patients was not negligible (18/126, 14%, Figure 4B), and the landmark analysis suggests that the presence of AS at baseline is indeed a major factor in the development of AVD progression in the long-term postoperative follow-up (Figure 2B,C and Figure 3B,C), with an alarming lifetime cumulative risk in young patients. Combined valvular disease may lead to paradoxical low flow and low gradient, resulting in more pronounced deterioration of cardiac function and higher mortality rates [34]. In such cases, combined aortic and mitral mechanical valve replacement might seem like a definitive solution to avoid future reoperations. However, increased surgical and cardiopulmonary bypass time, higher perioperative risks of organ dysfunction, increased anticoagulation requirements, lifestyle restrictions (e.g., career choices, pregnancy planning), and greater psychological burden are issues that deserve more clinician attention than the rate of AVD progression. While opting for mitral valve replacement in younger patients may delay reoperation to an older age—a stage associated with higher mortality risks from re-sternotomy—advances in surgical techniques (e.g., minimally invasive approaches) and transcatheter aortic valve implantation may tilt the risk–benefit balance in favor of future interventions, which could potentially outweigh the hazards of surgery.

## 5. Conclusions

This study highlights that baseline untreated mild AVD in patients undergoing mitral valve surgery is associated with a significant negative impact on long-term outcomes, especially mild AS. However, the included trials were single-center, retrospective research studies, underscoring the need for future large-scale, multicenter randomized controlled trials involving broader patient populations. Such research would more precisely identify high-risk subgroups prone to disease progression and elevated reoperation rates. For patients with baseline AS, investigations should focus on how variations in lesion characteristics (e.g., anatomical localization), hemodynamic profiles, and peak pressure gradients influence the progression of AS. Lastly, systematic postoperative follow-up is critical for AVD patients undergoing MVR to monitor disease trajectories and optimize clinical management.

## Figures and Tables

**Table 1:** Study characteristics of the articles included in the systematic reviews and meta-analysis.

Study	Country	Study Period	Study Type	AVD/NAVD	AS/AR	Mean Follow-Up Years	Primary and Endpoints	NOS
Vaturi 1999 [4]	Israel	1975–1992	Retrospective cohort	59/72	——	13 ± 7 years	Progression of AVD and reoperation rates in long-term follow-up	6
Choudhary 2001 [11]	India	1979–1997	Retrospective cohort	——	52/232	10.8 ± 3.7 years	Progression of AVD and reoperation rates in long-term follow-up	7
Namboodiri 2009 [5]	India	1994–1996	Retrospective cohort	96/194	17/69	11.98 ± 6.4 years	Progression of AVD and reoperation rates in long-term follow-up	5
Hwang 2014 [6]	Korea	1992–2010	Retrospective cohort	——	7/104	11.8 years	Reoperation rates in long-term follow-up	7
Kim 2018 [8]	Korea	1990–2015	Retrospective cohort	358/865	66/292	11.2 ± 7.3 years	Progression of AVD and reoperation rates in long-term follow-up	7
Kim 2023 [9]	Korea	1997–2015	Retrospective cohort	275/880	——	142.1 months	Progression of AVD and reoperation rates in long-term follow-up	7

**Table 2:** The clinical characteristics of the participants.

Variable	AVD	NAVD	*p*	RR/MD
Number of patients	618	999	/	/
Sex, female	438	699	0.704	0.365
Mean age, years	50.7 ± 13.4	50.1 ± 12.2	0.355	1.013 (0.949, 1.081)
Atrial fibrillation	556	912	0.373	0.986 (0.957, 1.016)
NYHA class ≥ 3	399	760	<0.001	0.849 (0.793, 0.908)
Associated diseases				
Diabetes mellitus	45	53	0.105	1.373 (0.935, 2.016)
Hypertension	36	81	0.085	0.718 (0.492, 1.050)
Coronary artery disease	17	30	0.772	0.916 (0.510, 1.647)

Clinical characteristics of participants in included studies. Abbreviations: AVD: aortic valve disease; NAVD: non-aortic valve disease.

## Data Availability

The raw data supporting the conclusions of this article will be made available by the authors, without undue reservation.

## Supplementary Materials

The following supporting information can be downloaded at: https://www.mdpi.com/article/10.3390/jcdd12110426/s1, PRISMA checklist is available in the Supplementary Materials.

## Abbreviations

The following abbreviations are used in this manuscript:

RHD	Rheumatic heart disease
RCT	Randomized controlled trials
AVD	Aortic valve disease
CI	Confidence intervals
HR	Hazard ratios
AVR	Aortic valve replacement
NAVD	Non-aortic valve disease
AS	Aortic stenosis
AR	Aortic regurgitation
